# Editorial: Contributing Factors to Renal Dysfunction: Fetal Programming, Hormones, and Epigenetic

**DOI:** 10.3389/fphys.2021.793730

**Published:** 2021-12-06

**Authors:** Guiomar Nascimento Gomes, Minolfa C. Prieto, Karina Thieme, Heloisa Della Coletta Francescato

**Affiliations:** ^1^Department of Physiology, Federal University of São Paulo, São Paulo, Brazil; ^2^Department of Physiology, Tulane University School of Medicine, New Orleans, LA, United States; ^3^Department of Physiology and Biophysics, University of São Paulo, São Paulo, Brazil; ^4^Department of Physiology, Faculty of Medicine of Ribeirão Preto, University of São Paulo, Ribeirao Preto, Brazil

**Keywords:** fetal programming, renal dysfunction, miRNA—microRNA, renin–angiotensin–aldosterone system, renal fibrosis

Over the past few decades, many advances led to a better understanding of the mechanisms involved in renal development and function. Even so, kidney disease is still one of the most prevalent complications in chronic conditions, including cardiovascular diseases, diabetes, and obesity. Thus, it is timely and essential to deepen knowledge about the underlying factors predisposing to renal dysfunction, such as fetal programming, epigenetic, hormonal, or nutritional interferences.

Recently, the interest in the link between *in utero* fetal stressors, epigenetics, and the risk of later in life diseases has surged. Evidence have demonstrated that early *in utero* maladaptation may have repercussion on health outcomes in adulthood. However, most of the involved mechanisms remain unclear, and developments are underway.

In this collection, an extensive review of recent evidence and discoveries highlighting contemporary understanding of this intriguing and sometimes controversial relationship.

The appraisal of fetal programming is introduced through the review article: “Programmed kidney disease in adults: importance of the fetal environment” (Argeri et al.). The authors describe the impact of adverse conditions during pregnancy on the fetal kidneys, focusing on the mechanisms underlying epigenetic events, and the components/functions of the renin–angiotensin–aldosterone system (RAAS). Still, in the field of fetal programming, Barros Sene et al. present the role of specific miRNAs involved in molecular modulations during nephrogenesis. The kidneys from low-protein-intake offspring on the 17^th^ gestational day reveal changes in miRNAs and the molecular pathways, reducing the reciprocal interaction between the metanephric cap and the ureter bud. Additionally, the renal structural and functional modifications found in the fetal programming model of protein restriction during gestation and lactation are detailed (Lamana et al.).

Epigenetics was also explored in its relationship with the role of extracellular vesicles in the context of tuberous sclerosis complex (TSC) disease. Tuberous sclerosis complex-associated renal phenotypes show considerable variability, adding another layer of complexity to the diagnosis and proper treatment. Thus, studying other mechanisms associated with this disease pathogenesis also offers new therapeutic avenues. In this sense, using mouse inner medullary collecting duct (mIMCD) cells with the Tsc1 (T1G cells) or Tsc2 (T2J cells) gene disrupted by CRISPR/CAS9 Kumar et al. demonstrated that miR-212-3p/mTORC1 and mIR-99a-5p/mTORC1 axis could be a novel therapeutic target or biomarker for TSC.

Besides epigenetics, new genetic and molecular aspects involved in the development of kidney disease are also discussed in this special issue. Epithelial–mesenchymal transition (EMT) is essential for the progression of kidney fibrosis. However, tubular epithelial cells (TEC) can first undergo partial EMT, secreting chemokines and cytokines to promote fibrogenesis and inflammation, leading to kidney fibrosis. The review by Sheng and Zhuang emphasizes the most recent findings related to the process of “partial epithelial–mesenchymal transition (pEMT)” and its contribution to renal fibrogenesis, including cell cycle arrest, metabolic alternation of epithelial cells, infiltration of immune cells, epigenetic regulation, as well as new signaling pathways that mediate this altered epithelial–mesenchymal communication. Furthermore, the participation of the transcription factor Tisp40 in the process of the TEC pyroptosis after the renal ischemia-reperfusion injury is detailed by Xiao et al. Likewise, Li et al. showed that α-lipoic acid acts as a protective molecule in folic acid-induced kidney damage, reducing tubular cell death mainly by inhibiting ferropotosis.

Acute crystalline nephropathy is usually associated with damage to the tubulointerstitial compartment. Few studies have investigated glomerular injuries in this condition. De Araújo et al. unveil aspects of glomerular and tubulointerstitial injury in an experimental model of crystalline-induced acute kidney injury, with kidney function decline and reduction in podocyte markers, nephrin, and WT1, possibly leading to a podocyte loss.

The role of renal sympathetic nerves in hypertension has been explored in the last decades. However, the role of the renal afferent innervation is still poorly understood. In a well-designed study, the role of renal afferent nerves on GABAergic signaling on the paraventricular nucleus of the hypothalamus (PVN) was investigated in the 2-kidney 1-clip (2K1C) renovascular hypertensive rat model (Milanez et al.). The selective removal of renal sensory innervation enhanced GABAergic inputs into the PVN, which may have contributed to the decrease in blood pressure and sympathetic excitation, as well as improvement in renal function and proteinuria, as indicated previously.

Addressing the use of angiotensin-converting enzyme inhibitor or angiotensin receptor blocker, alone or in combination in arterial hypertension, a significant antiproteinuric effect in hypertensive individuals was observed. To better understand this effect, Corrêa et al. evaluated in 2K1C rats the result of the RAAS double blockade on the expression of filtration barrier components as well as components involved in proximal protein transport. The double blockade restored the expression of podocin, cubillum, and ClC5, likely related to the antiproteinuric effect. On the other hand, the double blockade of the RAAS, due to an exacerbated negative regulation of the active form of the alpha epithelial sodium channel (α-ENaC), lead to hyperkaliemia.

The role of AT1R-associated protein (ATRAP) on potassium transport in distal nephron was explored by Polidoro et al. This protein attenuates the inhibition of ROMK channels by angiotensin II in collecting duct cells, probably by decreasing c-Src activation and blocking ROMK internalization.

In sum, chronic kidney disease (CKD) is a worldwide public health problem with an increasing prevalence. Studies directed to understand better the events related to CKD are imperative with the development of strategies to minimize the progression of this disease. In this way, the present collection of articles brings relevant contributions to molecular and epigenetic mechanisms modulating nephrogenesis under the influence of the intrauterine environment and recent advances related to the pathophysiology of renal fibrosis. Also, the emergent experimental strategies carry a better understanding and in the management of arterial hypertension.

## Author Contributions

All listed authors contributed equally to the elaboration of the manuscript and approved its final format for publication.

## Conflict of Interest

The authors declare that the research was conducted in the absence of any commercial or financial relationships that could be construed as a potential conflict of interest.

## Publisher's Note

All claims expressed in this article are solely those of the authors and do not necessarily represent those of their affiliated organizations, or those of the publisher, the editors and the reviewers. Any product that may be evaluated in this article, or claim that may be made by its manufacturer, is not guaranteed or endorsed by the publisher.

